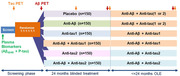# The Alzheimer’s Tau Platform (ATP): a Phase 2, combination amyloid and tau therapy clinical trial for early AD

**DOI:** 10.1002/alz.085111

**Published:** 2025-01-09

**Authors:** Adam L. Boxer, Reisa A Sperling, Paul S. S Aisen, Ronald C. Petersen, Michael C. Donohue, Rema Raman, Randall J. Bateman, Eden V. Barragan, Dorene M. Rentz, Joshua D Grill, Jason Karlawish, Robert A Rissman, Keith A Johnson, Alzheimer’s Clinical Trial Consortium (ACTC)

**Affiliations:** ^1^ Memory and Aging Center, UCSF Weill Institute for Neurosciences, San Francisco, CA USA; ^2^ Harvard Medical School, Cambridge, MA USA; ^3^ Alzheimer’s Therapeutic Research Institute, Keck School of Medicine, University of Southern California, San Diego, CA USA; ^4^ Department of Neurology, Mayo Clinic, Rochester, MN USA; ^5^ Alzheimer’s Therapeutic Research Institute, University of Southern California, San Diego, CA USA; ^6^ Washington University School of Medicine, St. Louis, MO USA; ^7^ University of California, San Francisco, San Francisco, CA USA; ^8^ Massachusetts General Hospital, Boston, MA USA; ^9^ Institute for Memory Impairments and Neurological Disorders, University of California, Irvine, Irvine, CA USA; ^10^ University of Pennsylvania, Philadelphia, PA USA; ^11^ University of Southern California, San Diego, CA USA; ^12^ Massachusetts General Hospital, Harvard Medical School, Boston, MA USA

## Abstract

**Background:**

Anti‐amyloid immunotherapies modestly slow disease progression in early symptomatic AD; addition of other therapeutic modalities may be necessary to achieve larger treatment effects. Therapies that directly target tau can potentially produce substantial clinical benefit because the accumulation of insoluble tau protein is strongly correlated with the progression of AD. Which tau therapies are likely to be efficacious, whether or not to combine them with anti‐amyloid therapies, and which individuals are most likely to benefit are important unresolved questions that would require multiple parallel design trials to answer. Platform (umbrella) trials test multiple therapies in the same trial, allowing for substantial savings in time, cost and participant burden.

**Method:**

ATP is a new NIH‐funded Phase 2 clinical trial platform that will be conducted as part of the AD Clinical Trials Consortium (ACTC) in partnership with industry and philanthropic groups. The goal is to investigate the effects of tau therapies on the biological hallmarks of AD including insoluble and soluble tau species, amyloid and other biomarkers of neurodegeneration. ATP will initially evaluate two tau therapies alone or in combination with an anti‐amyloid therapy. 900 participants with late preclinical or early prodromal AD (CDR 0 or 0.5) will be randomized to six arms for two years of blinded treatment, followed by an optional open label extension (Figure 1). At least 20 percent of participants will be from underrepresented groups. The primary endpoint is ^18^F MK6240 tau PET, with key secondary endpoints including plasma P‐tau and MTBR243‐tau, amyloid PET and clinical assessments (CDR‐SB, PACC5). Inclusion criteria are based on plasma P‐tau217, clinical status and regional ^18^F MK6240 uptake.

**Result:**

Two tau therapies and an anti‐amyloid therapy have been chosen for inclusion in ATP and startup is underway. The final design, choice of therapies and design as well as plans for inclusion of additional tau therapies after the trial begins and other details will be presented.

**Conclusion:**

The ATP is a six arm factorial design platform trial that is planned to begin in late 2024 or early 2025 that will support efficient development of tau and combination therapies for early, sporadic AD.